# Staged Reconstruction With a Reverse Latissimus Dorsi Flap for Late-Onset Lumbar Surgical Site Infection in a Previously Irradiated Field: A Case Report

**DOI:** 10.7759/cureus.116045

**Published:** 2026-09-10

**Authors:** Shun Suzawa, Yasuhito Yahara, Mineyuki Zukawa, Katsuhiko Kamei, Yoshiharu Kawaguchi

**Affiliations:** 1 Department of Orthopaedic Surgery, Faculty of Medicine, University of Toyama, Toyama, JPN

**Keywords:** lumbar spine reconstruction, lumbar spine surgery, radiation-associated soft tissue injury, reverse latissimus dorsi flap, surgical site infection

## Abstract

Surgical site infection (SSI) after spinal instrumentation surgery remains a challenging complication, particularly in patients with a history of radiotherapy. Radiation-associated skin and soft tissue injuries cause long-lasting impairment of vascularity and regenerative capacity, significantly compromising wound healing and infection control. When SSI occurs in radiation-affected tissue, wound closure becomes difficult, requiring complex reconstructive surgery. A 73-year-old man who had previously received radiotherapy to the lumbar region for malignant lymphoma at the age of 37 (in 1989) developed a late-onset lumbar SSI seven years after posterior lumbar interbody fusion at L3/4. Initial surgical debridement followed by primary wound closure failed, leading to wound dehiscence two weeks postoperatively. Considering the impaired biological regenerative activity of the irradiated tissues, repeated primary closure was avoided. Instead, staged management consisting of extensive debridement, negative pressure wound therapy (NPWT) with continuous local irrigation, and definitive reconstruction using a reverse latissimus dorsi musculocutaneous flap was performed. Although flap coverage was insufficient for the lower lumbar to sacral region, continued NPWT promoted tissue granulation, allowing successful split-thickness skin grafting. Complete wound closure was ultimately achieved. SSI in irradiated tissue may reflect impaired regenerative capacity in addition to the infectious process itself. A reverse latissimus dorsi musculocutaneous flap combined with NPWT and skin grafting enabled successful closure of a large tissue defect of the lumbar spine without infection recurrence at six-month follow-up.

## Introduction

Radiation therapy is well known to cause long-lasting and often irreversible damage to skin and soft tissue [1]. Radiation-associated skin and soft tissue injuries are characterized not only by superficial skin changes, such as tissue atrophy and fibrosis, but also by profound impairment of microvascular circulation, cellular regenerative capacity, and local immune function. Importantly, these effects may persist for decades after irradiation and can significantly compromise wound healing following subsequent surgical intervention [2,3].

When spinal surgery is performed within previously irradiated fields, postoperative complications, including infection, wound dehiscence, and nonhealing wounds, may occur, although the reported risk varies depending on the radiation technique, dose, timing, and patient population [4]. In such cases, conventional strategies such as debridement and primary wound closure often fail, as they do not address the fundamentally impaired biological environment of irradiated tissues. Negative pressure wound therapy (NPWT) can facilitate wound management by reducing edema and exudate and promoting granulation tissue formation, thereby preparing the wound bed for definitive reconstruction. However, NPWT alone may be insufficient in the presence of extensive soft tissue defects and deep dead space. Thus, reconstruction using well-vascularized tissue from nonirradiated areas is required to restore a favorable healing environment.

The reverse latissimus dorsi musculocutaneous flap, based on posterior intercostal perforators, has been described as a valuable option for coverage of the lumbar spine [5,6]. By introducing healthy, highly vascularized tissue into compromised wounds, this technique can help obliterate dead space and cover exposed spinal structures, potentially contributing to infection control and wound healing [7-9].

Here, we report a challenging case of late-onset lumbar surgical site infection (SSI) occurring seven years after posterior lumbar interbody fusion (PLIF) in a patient with a history of lumbar radiotherapy for malignant lymphoma. This case illustrates the potential long-term impact of radiation-associated tissue injury on wound-healing capacity and highlights the potential value of a staged approach combining infection management, NPWT, and reverse latissimus dorsi flap reconstruction for complex spinal wounds.

## Case presentation

A 73-year-old man presented to our hospital with a one-week history of fever and severe low back pain. He had undergone PLIF at the L3/4 level seven years earlier, complicated by right lower extremity paresis (manual muscle testing grade 1), which showed no worsening at presentation. On admission, his body temperature was 39.6°C, and the lumbar skin showed dark-red pigmentation with marked swelling (Figure 1A) and tenderness, without wound drainage. Laboratory investigations revealed markedly elevated inflammatory markers, with a white blood cell count of 11,010/μL and a C-reactive protein level of 22.81 mg/dL. The total protein and serum albumin levels were 7.1 g/dL and 2.7 g/dL, respectively, while the blood glucose level was 109 mg/dL and glycated hemoglobin (HbA1c) was 5.5%. The patient had no history of diabetes or other conditions predisposing to infection. X-ray imaging confirmed solid bone fusion at the L3/4 level (Figure 1B). MRI demonstrated abscess formation posterior to the surgical site, with no obvious loosening of the instrumentation (Figure 1C, Figure 1D). *Staphylococcus lugdunensis* was isolated from puncture fluid obtained from the lumbar spine, whereas blood cultures obtained at the initial presentation were negative. Antimicrobial susceptibility testing showed that the isolate was susceptible to the tested β-lactam agents and vancomycin. Notably, the patient had received radiotherapy to the lumbar region for malignant lymphoma at age 37 (in 1989); the exact radiation dose was unknown. Based on these findings, the patient was diagnosed with late-onset lumbar SSI, with chronic radiation-associated skin and soft tissue injury considered a potential contributing factor.

**Figure 1:**
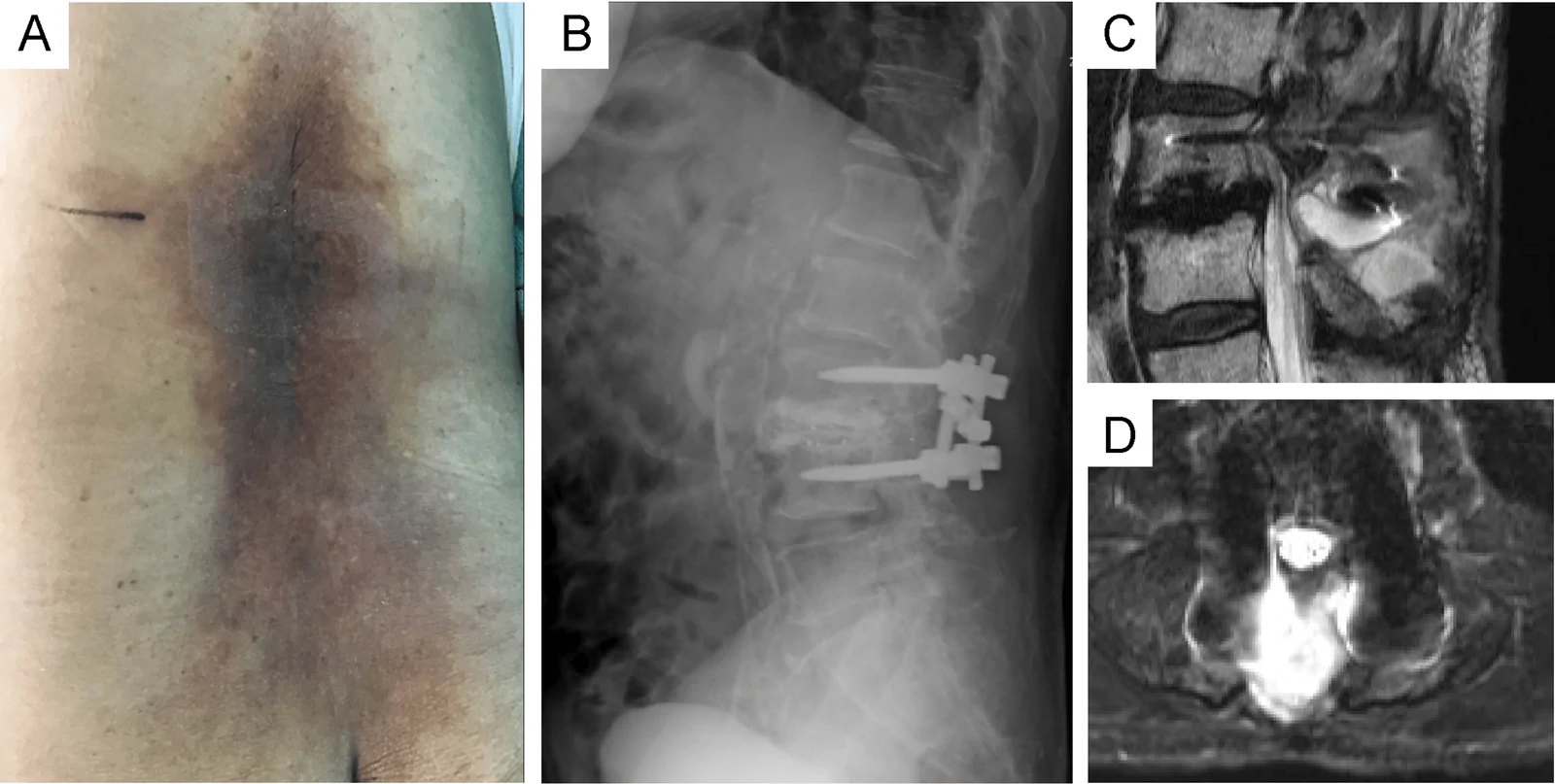
Preoperative findings of lumbar surgical site infection (A) Lumbar skin showing dark-red discoloration, swelling, and radiation-associated hyperpigmentation and scarring. (B) Radiograph showing solid bone fusion at L3/4 with instrumentation. (C, D) Sagittal and axial T2-weighted MRI images showing an abscess within the paraspinal muscle posterior to the surgical site.

Initial surgical management

Surgical debridement was performed the day after admission. Intraoperatively, the subcutaneous tissue and paraspinal muscles were severely fibrotic and poorly vascularized, suggestive of chronic radiation-associated tissue injury. Considering the achieved bone fusion at L3/4, the instrumentation was removed to facilitate infection control. After thorough debridement, primary wound closure was attempted, and closed suction drainage was placed. Given the concern for a deep postoperative spinal infection, empirical piperacillin/tazobactam was started after culture specimens were obtained by needle aspiration at the initial evaluation, without waiting for the definitive culture and susceptibility results, and was subsequently changed to cefepime after *Staphylococcus lugdunensis* was identified. However, wound dehiscence occurred two weeks after surgery (Figure 2A).

**Figure 2:**
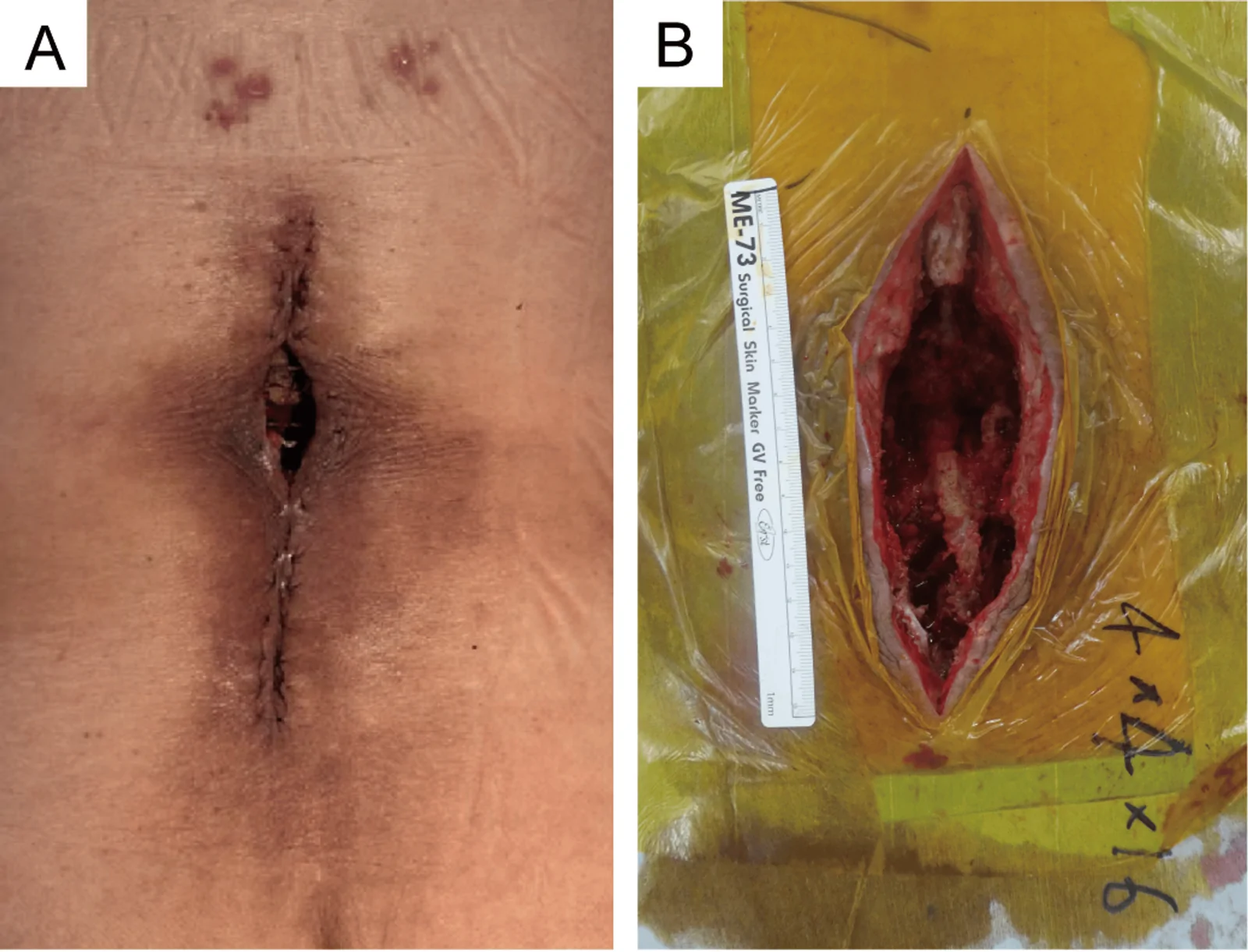
Wound findings after wound dehiscence and second-stage debridement (A) Wound dehiscence at the surgical site. (B) Appearance immediately after debridement performed during the second surgery, showing a deep soft-tissue defect measuring approximately 4 × 4 × 16 cm.

Second-stage strategy: infection control without closure

Considering the compromised condition of the local soft tissues and the failure of initial primary closure, we judged that repeat primary closure would likely result in the same unfavorable outcome. Therefore, during the second surgery, only debridement was performed, and the wound, with a resulting soft-tissue defect measuring approximately 4 × 4 × 16 cm, was intentionally left open (Figure 2B). NPWT with continuous irrigation was initiated at a negative pressure of −50 mmHg, with normal saline administered continuously at 500 mL/day. The dressing was changed once weekly. This treatment was continued for two weeks, during which local inflammatory findings gradually subsided, and the wound bed became suitable for subsequent reconstruction. During this period, *Corynebacterium striatum* was additionally isolated. Antimicrobial susceptibility testing demonstrated resistance to multiple β-lactam agents, including cefepime, whereas susceptibility to vancomycin was retained. The antimicrobial regimen was subsequently changed to sulbactam/ampicillin and vancomycin. Antimicrobial therapy, including subsequent oral treatment, was continued for a total of three months from the initial presentation.

Reconstruction with a reverse latissimus dorsi flap

Following repeated debridement and NPWT, the local wound condition improved sufficiently to allow definitive reconstruction. At this point, the residual wound was a deep soft-tissue defect with exposed spinous processes and laminae and exposed dura mater at its base (Figure 3A). A reverse latissimus dorsi musculocutaneous flap, based on posterior intercostal perforators, was selected to introduce well-vascularized, nonirradiated tissue into the compromised lumbar wound (Figure 3B, Figure 3C). The flap showed excellent perfusion and engraftment, successfully covering the upper lumbar defect (Figure 3D, Figure 3E).

**Figure 3:**
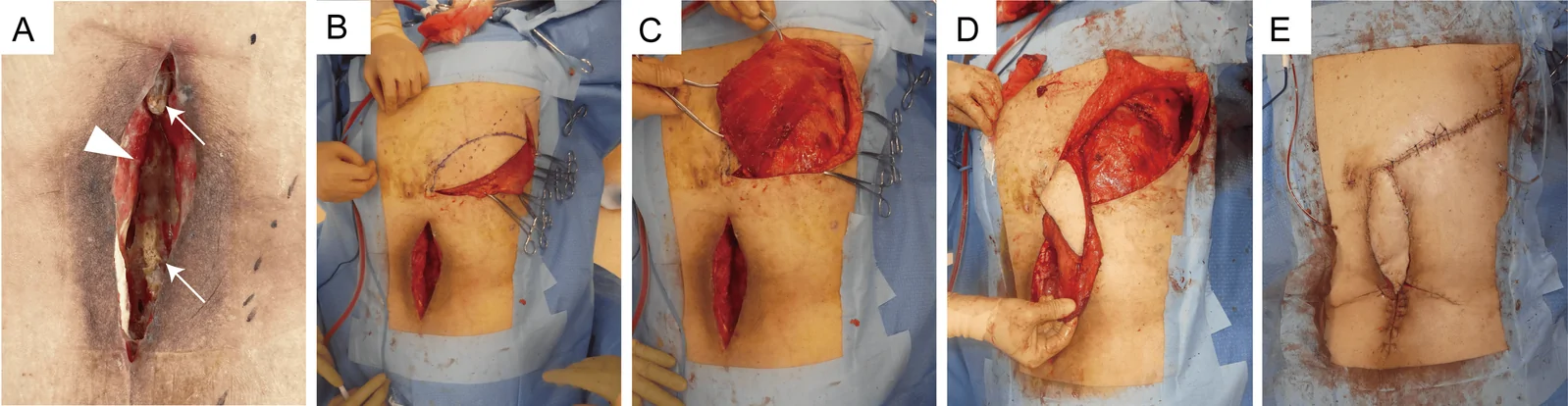
Reverse latissimus dorsi flap reconstruction (A) Appearance of the residual wound after negative pressure wound therapy, immediately before definitive reconstruction, showing exposed spinous processes (white arrows) and exposed dura mater (white arrowhead). (B, C) Design and elevation of the reverse latissimus dorsi musculocutaneous flap. (D) Transposition of the flap into the lumbar defect. (E) Appearance after flap inset and closure.

However, the distal portion of the flap covering the lower lumbar to sacral region subsequently developed partial necrosis with wound dehiscence over the sacral area, and additional debridement was performed (Figure 4A). The underlying muscle component of the flap remained viable, maintaining obliteration of the deep dead space and continued coverage of the exposed spinal structures. To address the remaining defect, NPWT was continued to promote granulation tissue formation in the uncovered area (Figure 4B). Once sufficient granulation tissue had formed, the residual defect, measuring approximately 8 × 4 cm, was covered with a split-thickness skin graft harvested from the abdomen (Figure 4C). Complete wound closure was ultimately achieved. At the six-month postoperative follow-up, the wound remained completely healed with no evidence of infection recurrence (Figure 4D).

**Figure 4:**
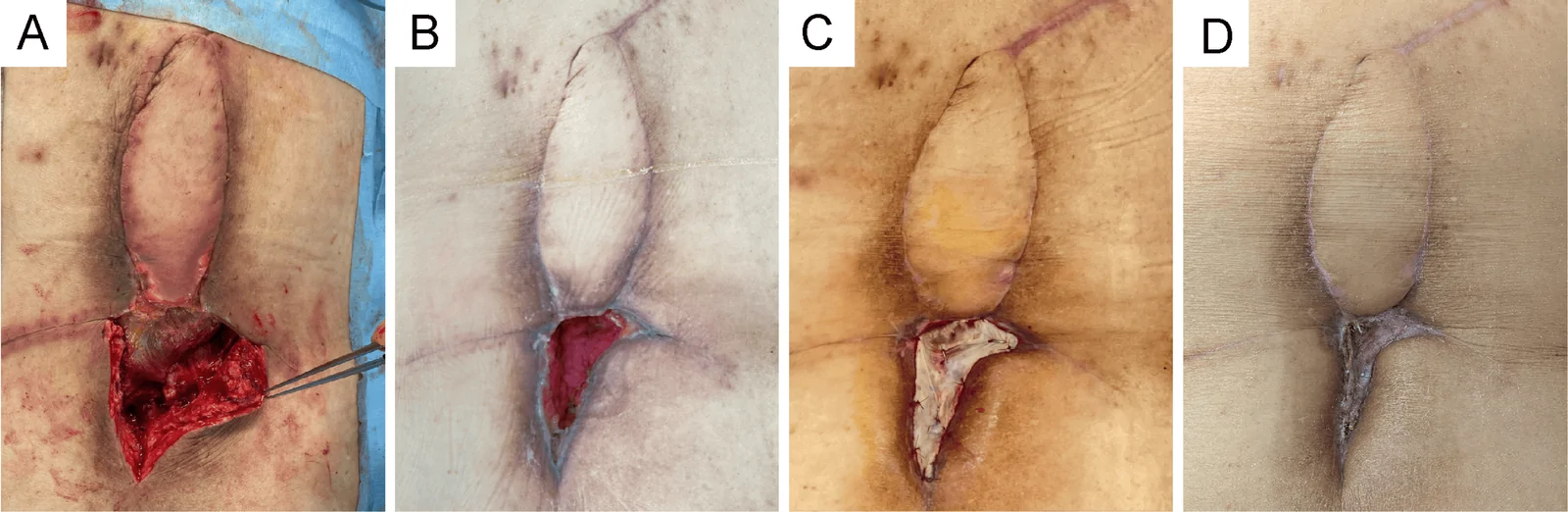
Wound management after partial flap necrosis (A) Partial flap necrosis with dehiscence over the sacral area, requiring additional debridement. (B) Well-formed granulation tissue prior to skin grafting. (C) Appearance immediately after split-thickness skin grafting of the residual defect (approximately 8 × 4 cm). (D) Final appearance after complete wound healing at six-month follow-up.

## Discussion

Recent studies have reported comparable rates of wound complications between patients with and without a history of radiotherapy [10,11], indicating that the association between previous radiotherapy and postoperative wound complications is not uniform across clinical settings. However, these findings largely reflect modern radiotherapy techniques, which allow more precise optimization of the target volume and radiation dose. In contrast, radiotherapy for malignant lymphoma in the 1980s often involved irradiation of relatively extensive areas of surrounding healthy tissue [12]. In such patients, radiation-associated soft tissue injury may remain clinically silent for several decades before becoming apparent [13]. Although irradiated tissue may appear macroscopically normal, its regenerative capacity and microvascular network can remain compromised, potentially contributing to impaired wound healing and increased susceptibility to wound complications following surgical intervention. Delanian et al. proposed the concept of the radiation-induced fibroatrophic process, in which chronic inflammation, persistent reactive oxygen species production, endothelial injury, and progressive fibrosis contribute to tissue damage [14]. Furthermore, Marx et al. demonstrated that radiation-induced endarteritis obliterans results in microvascular compromise, leading to hypoxia, hypovascularity, and hypocellularity of the affected tissues [15].

In the present case, the patient underwent lumbar irradiation in 1989 for malignant lymphoma. The chronic skin and soft tissue changes, accompanied by extensive tissue fibrosis, were considered potential contributing factors to the refractory course of the lumbar SSI. However, hypoalbuminemia at presentation may also have contributed to impaired wound healing. Therefore, the refractory wound course cannot be attributed solely to previous radiotherapy, and a direct causal relationship between radiotherapy and the development of SSI cannot be established in this case. In addition, the mechanism underlying the delayed onset of SSI seven years after PLIF remains unclear. *Staphylococcus lugdunensis* was initially isolated from the lumbar lesion, and *Corynebacterium striatum* was subsequently isolated during the clinical course. However, the clinical significance of the latter isolate was uncertain. Therefore, the precise source and mechanism of the delayed SSI could not be determined in this case.

This clinical course illustrates why a staged surgical strategy, rather than repeated attempts at primary closure, was necessary in this setting. After the initial closure failed, repeated debridement and antimicrobial therapy were followed by NPWT with continuous irrigation to improve the local wound condition before flap reconstruction. This approach reflects growing evidence supporting NPWT as a bridge to definitive closure in complex spinal wounds: NPWT can stimulate granulation tissue formation, which facilitates subsequent flap placement and may reduce the need for more extensive reconstructive procedures [16]. Similarly, West et al. described a protocol of serial debridement and instillation of NPWT followed by definitive closure with muscle flaps in patients with infected spinal hardware [17]. In the present case, despite antimicrobial therapy and repeated surgical debridement, definitive wound healing could not be achieved because a substantial deep dead space with exposed lamina and dura mater persisted. Thus, infection control alone was insufficient to achieve definitive wound healing, and reconstruction with well-vascularized tissue was required once the local wound condition had sufficiently improved.

The latissimus dorsi flap provides a large volume of well-vascularized tissue and has long been used for spinal reconstruction. In 1980, Bostwick et al. first described its reverse form, demonstrating that the muscle is supplied not only by the thoracodorsal vessels but also by perforating intercostal vessels [5]. Stevenson et al. subsequently showed, through anatomical and clinical studies, that the posterior branches of the ninth to eleventh intercostal arteries provide reliable perfusion to the distal portion of the muscle, allowing safe elevation of a reverse latissimus dorsi flap [18]. Watanabe et al. further demonstrated the presence of multiple vascular territories within the latissimus dorsi muscle connected by choke vessels, providing an anatomical explanation for the reliability of reverse flap perfusion [19].

Collectively, these studies established the vascular basis of the reverse latissimus dorsi flap and expanded its application to the reconstruction of complex lumbosacral defects. In addition, transfer of vascularized muscle tissue improves local perfusion, enhances oxygen delivery, and facilitates antibiotic penetration into poorly vascularized tissues, all of which may contribute to infection control and wound healing [7-9]. de Fontaine et al. reported favorable outcomes using a reverse turnover latissimus dorsi flap for complex lumbosacral defects associated with dural exposure and deep dead space, highlighting its value as a reconstructive option in these challenging situations [20].

Although partial flap necrosis and wound dehiscence occurred over the distal sacral area, the reverse latissimus dorsi flap successfully obliterated the deep dead space and provided stable coverage of the exposed spinal structures. The residual superficial defect was subsequently managed with NPWT until sufficient granulation tissue had formed, followed by split-thickness skin grafting. This experience highlights the importance of designing the flap to provide adequate tissue volume for dead-space obliteration while tailoring the skin paddle to the anticipated cutaneous defect.

This report has certain limitations. The radiation dose and the details of the chemotherapy regimen administered in 1989 could not be ascertained, and the diagnosis of radiation-associated soft tissue injury was based on the clinical course and intraoperative macroscopic findings rather than histopathological confirmation. In addition, the six-month postoperative follow-up period is relatively short, considering that such injury may remain clinically latent for several decades.

## Conclusions

This case suggests that impaired regenerative capacity in previously irradiated tissue may complicate the management of SSI in addition to the infectious process itself. In this patient, a staged approach incorporating debridement, implant removal, continuous irrigation, NPWT, reverse latissimus dorsi musculocutaneous flap reconstruction, and subsequent split-thickness skin grafting resulted in successful wound closure without infection recurrence at the six-month follow-up. This approach may represent a useful reconstructive option for selected complex lumbar wounds in previously irradiated fields; however, further clinical experience is required to determine its broader applicability.
